# Antimicrobial and toxicological activities of five medicinal plant species from Cameroon Traditional Medicine

**DOI:** 10.1186/1472-6882-11-70

**Published:** 2011-08-25

**Authors:** Jules CN Assob, Henri LF Kamga, Dickson S Nsagha, Anna L Njunda, Peter F Nde, Emmanuel A Asongalem, Abdel J Njouendou, Bertrand Sandjon, Veronique B Penlap

**Affiliations:** 1 Medicine Programme, Department of Biomedical Sciences, Faculty of Health Sciences Laboratory, University of Buea. P.O.Box 63, Buea, Cameroon; 2 Department of Medical Laboratory Sciences, Faculty of Health Sciences, University of Buea. P.O.Box 63, Buea, Cameroon; 3 Department of Public Health and Hygiene, Faculty of Health Sciences, University of Buea. P.O.Box 63, Buea, Cameroon; 4 Department of Biochemistry, Faculty of Science, University of Buea, P.O.Box 63, Buea, Cameroon; 5 Laboratoire Phytorica, P.O.Box 12521 Bonapriso, Douala. Cameroon; 6 Laboratoire de Microbiologie Appliquée et de Pharmacologie Moléculaire, Département de Biochimie, Université de Yaoundé I. P.O. Box 812 Yaoundé, Cameroon

**Keywords:** *Phyllanthus muellerianus*, *Piptadeniastum africana*, antimicrobial, acute toxicity, kidney and liver function tests, Cameroon Traditional Medicine

## Abstract

**Background:**

Infectious diseases caused by multiresistant microbial strains are on the increase. Fighting these diseases with natural products may be more efficacious. The aim of this study was to investigate the *in vitro *antimicrobial activity of methanolic, ethylacetate (EtOAc) and hexanic fractions of five Cameroonian medicinal plants (*Piptadeniastum africana*, *Cissus aralioides, Hileria latifolia, Phyllanthus muellerianus *and *Gladiolus gregasius) *against 10 pathogenic microorganisms of the urogenital and gastrointestinal tracts.

**Methods:**

The fractions were screened for their chemical composition and *in vivo *acute toxicity was carried out on the most active extracts in order to assess their inhibitory selectivity.

The agar well-diffusion and the micro dilution methods were used for the determination of the inhibition diameters (ID) and Minimum inhibitory concentrations (MIC) respectively on 8 bacterial species including two Gram positive species (*Staphylococcus aureus, Enterococcus faecalis)*, and six Gram negative *(Escherichia coli, Klebsiella pneumoniae, Pseudomonas aeruginosa, Proteus mirabilis, Shigella flexneri, Salmonella typhi) *and two fungal isolates (*Candida albicans, Candida krusei)*. The chemical composition was done according to Harbone (1976), the acute toxicity evaluation according to WHO protocol and the hepatic as well as serum parameters measured to assess liver and kidney functions.

**Results:**

The chemical components of each plant's extract varied according to the solvent used, and they were found to contain alkaloids, flavonoids, polyphenols, triterpens, sterols, tannins, coumarins, glycosides, cardiac glycosides and reducing sugars. The methanolic and ethylacetate extracts of *Phyllanthus muellerianus *and *Piptadeniastum africana *presented the highest antimicrobial activities against all tested microorganisms with ID varying from 8 to 26 mm and MIC from 2.5 to 0.31 mg/ml. The *in vivo *acute toxicity study carried out on the methanolic extracts of *Phyllanthus muellerianus *and *Piptadeniastrum africana *indicated that these two plants were not toxic. At the dose of 4 g/kg body weight, kidney and liver function tests indicated that these two medicinal plants induced no adverse effect on these organs.

**Conclusion:**

These results showed that, all these plant's extracts can be used as antimicrobial phytomedicines which can be therapeutically used against infections caused by multiresistant agents.

Phyllanthus muellerianus, Piptadeniastum africana, antimicrobial, acute toxicity, kidney and liver function tests, Cameroon Traditional Medicine

## Background

Infectious diseases are considered a major threat to human health, because of the unavailability of vaccines or limited chemotherapy. They account for approximately one half of all deaths in tropical countries [1]. Infectious diseases which ranked 5^th ^in 1981, became the 3^rd ^leading cause of death in 1992, with an increase of 58% [2]. Most of the current antibiotics have considerable limitations in terms of antimicrobial spectrum, side effects, and their widespread overuse has led to increasing clinical resistance of previously sensitive microorganisms and to the occurrence of uncommon infections [3]. Historically plants have provided a good source of anti-infective agents and the search for plants with antimicrobial activities has increasingly gained importance during recent years. With the advent of ever-increasing resistant bacteria and fungi strains, there has been a corresponding rise in the universal demand for natural antimicrobial therapeutics. Herbal medicines have widely been used and now form an integral part of the primary health care in many countries. They may constitute a reservoir of new antimicrobial substances to be discovered. Use of herbal medicines in Africa represents a long history of human interactions with the environment. Plants used for traditional medicine contain a wide range of substances that can be used to treat chronic as well as acute infectious diseases as stipulated by Diallo *et al. *[4]. In Cameroon, many plant species are used as traditional medicine to treat infectious diseases, and several interesting openings have originated for further inquiry following *in vitro *antimicrobial activity evaluation [5]. A previous epidemiological study on the characterization of urogenital tract microbial agents amongst women in the South West Region of Cameroon, revealed the presence of numerous multiresistant strains to currently used antibiotics in the treatment of urogenital tract infections [6]. Fighting local infections with natural products will be more advantageous and affordable to most patients. Our study was hence based on five selected medicinal plants described by Adjanohoun *et al. *[7] as traditional medication for various infectious ailments. The aim of this study was to evaluate the *in vitro *antimicrobial properties of methanolic, ethylacetate (EtOAc) and hexanic extracts of these five medicinal plants against eight Gram positive and Gram negative multiresistant bacteria species and two *Candida *isolates as well as to evaluate their *in vivo *acute toxicity on albino rats. The selection of these plants was based on their use in traditional medicine to cure current infectious diseases including urogenital tract infections [6].

## Methods

### Plants extraction and phytochemistry

Fresh plant parts were collected in August 2007. The medicinal plants used included the leaves of *Piptadeniastum africana *(Hook. f.) Bren. *(Mimosaceae)*, the leafy liana of *Cissus aralioides *(WELW EX BAKER) (*Vitaceae)*, the leaves of *Hileria latifolia *(Lam.) H.Walt *(Phytolacaceae)*, the stem barks of *Phyllanthus muellerianus *(O.Ktze) Exel. (*Euphorbiaceae*) and the bulbs of *Gladiolus gregasius *BAKER *(Iridaceae). Piptadeniastum africana *leaves were collected from Melong (Littoral Region of Cameroon) whereas others plants materials were collected from Dschang (West Region of Cameroon). These plants were identified at the Cameroon National Herbarium in Yaoundé. Fresh materials were dried at room temperature for about 3 weeks and crushed. Fractionation was done by macerating the powder obtained above into 6 L of hexane for two days. After filtration, the filtrate was evaporated to dryness under reduced pressure using a rotary vacuum evaporator. The resulting residues were successively macerated into the same volume (6 L) of ethylacetate (EtOAc) and methanol (MeOH). The extraction yield were evaluated (Table 1) and the dried crude extracts were stored at +4°C. The Phytochemical screening of the crude extracts was carried out using standard methods described elsewhere [8,9]. Each plant extract was screened for the presence of different classes of compounds including alkaloids, flavonoids, polyphenols, sterols, triterpenes, coumarins, anthraquinones, tannins, anthocyanins, saponins, glycosides.

**Table 1:** Extraction yield (%) and results of phytochemical screening of various medicinal plant's extracts

Plant Material	Solvent	Extraction yield (%)	Alk	Tri	St	Ant	Fla	Pol	Sap	Tan	Cou	Gly	CG	RS
***Piptadeniastum africana *(Leaves)**	**HEX**	0.60	+++	+++	++	-	-	-	-	-	+	+	-	-
	**ETA**	0.15	++	+++	++	-	-	-	-	+	+	+	+	+
	**MEOH**	1.75	-	+	+	-	++	++	-	+	++	++	++	++
***Cissus aralioides *(Leaves)**	**HEX**	0.26	++	++	++	-	-	-	-	+	+	+	+	+
	**ETA**	0.66	++	++	++	-	-	-	-	+	+	+	+	+
	**MEOH**	0.65	+	+	+	-	-	-	-		++	++	++	++
***Hileria latifolia *(Leaves)**	**HEX**	0.56	-	++	++	-	-	-	-	-	+	-	-	-
	**ETA**	1.55	-	++	++	-	-	-	-	-	+	-	-	-
	**MEOH**	4.05	-	+	+	-	-	-	-	-	+++	++	+++	+++
***Phyllanthus muellerianus *(stem bark)**	**HEX**	0.34	-	++	++	++	-	-	-	-	-	+	+	-
	**ETA**	0.53	-	-	-	-					+	+	+	+
	**MEOH**	1.81	+	+	+	++	++	++	-	-	+	+	+	+
***Gladiolus gregasius Baker *(Bulbs)**	**HEX**	0.71	++	++	++	++	-	-	-	-	-	+	+	-
	**ETA**	0.73	++	++	++	++	-	-	-	-	-	+	+	++
	**MEOH**	8.95	+	+	+	+	-	-	++	+	-	++	++	++

**Key: **HEX: hexane; ETA: Ethylacetate; MEOH: Methanol; Alk: Alkaloids; Tri: Triterpens; St: Sterols; Ant: Anthraquinons; Fla: Flavonoids; Pol: Polyphenols; Sap: Saponins; Tan: Tannins; Cou: Coumarins; Gly: Glycosides; CG: Cardiac glycosides; RS: Reducing sugarss

+++: abundantly found; ++: averagely found; +: trace

### Antimicrobial tests

Hexane, EtOAc and methanol extracts of the 5 medicinal plants were tested against a total of 8 bacterial species including two Gram positive species (*Staphylococcus aureus, Enterococcus faecalis)*, and 6 Gram negative *(Escherichia coli, Klebsiella pneumonia, Pseudomonas aeruginosa, Proteus mirabilis, Shigella flexneri, Salmonella typhi) *and 2 fungal isolates (*Candida albicans, Candida krusei) *from urogenital and GIT specimens obtained at the Buea Regional Hospital in Cameroon [6]. These isolates were characterized morphologically and biochemically using API galleries [6].

The susceptibility tests were performed using the agar well diffusion method as previously described [10-12]. Stock cultures were maintained at 4°C on Nutrient Agar (Oxoid, England) slopes. Active cultures for experiments were prepared by transferring a loopful of cells from the stock cultures to test tubes of Mueller-Hinton broth (Oxoid, England) for bacteria species and Sabouraud dextrose broth (Oxoid, England) for *Candida *species. They were incubated without agitation for 24 hrs at 37°C. The cultures were diluted with distilled water to achieve an optical density corresponding to 2.0 × 10^6 ^colony forming units per ml (CFU/ml) for bacteria species and 2.0 × 10^5^CFU/ml for *Candida *species. The medium was punched with six millimeters diameter wells and filled with 120 μl of the test sample. The concentration of the extracts employed was 50 mg/ml, prepared in 10% v/v aqueous Dimethyl Sulfoxide (DMSO). Simultaneously, Gentamycin (Sigma, USA) and Nystatin (Sigma, USA) were used as positive controls at a concentration of 0.2 mg/ml for bacteria and yeast cells respectively with 10% v/v aqueous/DMSO as negative control. The plates were incubated at 37°C for 24 hours, and inhibition zones formed around the wells were measured (mm) using a caliper. All tests were done in duplicate and the results were recorded as the mean diameter of the zones of growth inhibition surrounding the discs.

The Minimum Inhibitory Concentration (MIC) was determined by broth microdilution technique using 96-well plates [10,3]. Culture media were supplemented with 5% glucose and 1% phenol red end point indicator. After filling each well with 100 μL of broth, dry test samples previously diluted in DMSO were prepared to make a final concentration of 720 mg/ml. These solutions (100 μl) were added to the first well of each microtiter line. Successive dilutions were done by transferring the mixture/dilution (100 μl) from the first to the eleventh well. An aliquot (100 μl) was discarded from the eleventh well. The twelfth well served as control since no sample (extract, or reference antibiotics) was added in it. The microbial suspension (100 μl at 2.0 × 10^6 ^CFU/ml for bacteria species and 2.0 × 10^5^cells/ml for *Candida *species), obtained from an overnight growth was added to each well. The final concentration of the extracts used to evaluate the antimicrobial activity ranged from 160 to 0.15 mg/ml and reference drugs from 0.960 to 0.003 mg/ml. Tests were incubated aerobically at 37°C for 24 hours before being read. The end point was made by visual observation of growth. This was revealed by a colour change indicator from red to yellow. The MIC was considered as the lowest sample's concentration that prevented visible growth or changed in color from red to yellow due to the formation of acidic metabolites corresponding to microbial growth.

### Toxicity study

#### Experimental animals

Adult Wistar albino rats (120 - 140 g); were bred at the animal breeding house of Phytorica Lab in Douala. They were all clinically healthy and maintained in standard environmental conditions of temperature (28.0 ± 2°C). They were fed a standard diet and tap water *ad libitum. *The bioassay was conducted in accordance with the internationally accepted principles described by WHO [13] for laboratory use and care. Rats were deprived of food but not of water 12 h prior to administration of the test substance. Ethical clearance for animal experimentation was obtained from the Faculty of Health Sciences Institutional Review Board (Ref. No.: 2010/003/UB/FHS/IRB) at the University of Buea, Cameroon.

#### Acute toxicity

Acute toxicity was evaluated on extracts showing important antimicrobial properties; they were selected based on their MIC and the spectrum of antimicrobial activities. For each tested extract the procedure was as follow: The animals were separated in six groups of five males and five females including one control (group l) and five treated (groups 2-6). The treated groups (2-6) received 4; 8; 12; 16; 20 g/kg of body weight (bw) each respectively of oral single dose of each extract. The control group received tap water and or 20% aqueous DMSO at an equivalent volume. Observations were made and recorded systematically after 1 and 2 hours of extract administration. The visual observations included changes in respiratory pattern, motility and skin sensitivity, diarrhea, behavioral pattern, weight gain, food and water consumption. The number of survivors was noted after 48 hours and these were then maintained for a further 5 days, after which they were sacrificed by decapitation through an incision on the jugular vein to collect blood; sera were obtained after blood clotting and centrifugation at 3000 rpm for 10 min and used for biochemical analyses. The liver was also excised, rinsed in ice-normal saline solution and 20% (W/V) liver homogenates were prepared in a 0.1M Tris-HCl (pH 7.4) buffer solution. After centrifugation at 3000 rpm for 20 min the supernatant was separated and used for biochemical analysis. The medium lethal dose (LD_50_) was determined using the Behrens and Karber method [14]. Evaluation of the toxicity degree was based on previously described methods [15-17].

#### Biochemical estimation

Sera were assayed for liver function tests (aspartate aminotransferase (ASAT), alanine aminotransferase (ALAT) [18]), and kidney function tests (creatinin and urea [19]). Liver homogenates were assayed for aspartate aminotransferase (ASAT), alanine aminotransferase (ALAT) [18], malondialdehyde (MDA) [20] and glutathion (GSH) [21].

### Statistical analysis

One way analysis of variance (ANOVA) was applied for determining the statistical significance in various markers level between the control and the tested group. The level of significance was set at 0.05 and 0.01 [22].

## Results

### Phytochemical screening

Results presented in Table 1 below, show that all plant's extracts presented differences in phytochemical composition. Alkaloids, triterpens, sterols, flavonoids, tannins, coumarins, anthraquinons and reducing sugars were the main classes of compounds encountered in these plants. However, anthraquinones were not present in all the fractions of *P. africana, C. aralioides *and *H. latifolia. *The hexanic, ethylacetate and methanolic extracts presented a differential composition of the classes of chemical compounds tested positive in these plants. Alkaloids, triterpens, sterols were abundantly found in hexane and ethylacetate extracts, whereas flavonoids, anthraquinons were averagely present in ethylacetate and methanolic fractions. The fractions of *Piptadeniastrum africana *were all found to contain triterpens, sterols, tannins, coumarins, and glycosides. The fractions (hexanic, ethylacetate and methanolic extracts) of *Cissus aralioides *were tested positive to alkaloids, triterpens, sterols, tannins, coumarins, glycosides and cardiac glycosides, but the ethylacetate and methanolic extracts of this plant further contained reducing sugars. *Hileria latifolia *fractions were all tested positive to triterpens, sterols and coumarins, but in addition saponins, glycosides and reducing sugars were found only in the methanolic fraction. The hexanic, ethylacetate and methanolic extracts of *Phyllanthus muellerianus *(leaves) all contained triterpens, sterols, anthraquinons, coumarins, glycosides, cardiac glycosides and reducing sugars. The chemical composition of the 3 fractions of the bulbs of *Gladiolus gregasius *Baker showed that they all contained alcaloids, triterpens, sterols, anthraquinones, glycosides and cardiac glycoside, but reducing sugars and saponins were further present in the methanolic fraction.

### Antimicrobial activity

All plant extracts exhibited antibacterial and anticandidal activities. The ID and MIC obtained against Gram negative, Gram positive bacteria species, and yeast strains (*C. albicans, C. krusei) *are shown in Tables 2 and 3 respectively. Generally for all tested medicinal plants, the hexanic extract was poorly active than the ethylacetate and methanolic extracts except for *Gladiolus gregasius*. The ethylacetate and methanolic extracts of *Piptadeniastum africana *induced important inhibitory activities on tested Gram negative and Gram positive microorganisms with inhibition zone diameters (ID) varying from 8 to 26 mm and MIC from 2.5 to 0.31 mg/ml. The highest IDs (26 mm) were obtained with *S. flexneri *and *S. typhi *which are Gram negative strains, whereas the most sensitive Gram positive were *S. aureus *and *Candida albicans, (ID = 25 and MIC = 0.31 *mg/ml) against the methanolic extract. The ethylacetate fraction was less active when compared to the methanolic (with ID = 14 mm, MIC = 0.31 mg/ml on *S. aureus *and *Candida albican*s). In general the most important inhibition obtained was the methanolic fraction followed by the ethylacetate, and the lowest being the hexanic fraction which on many Gram negative microbial agents induced little or no inhibition (table 3).

**Table 2:** Inhibition zone diameters (ID in mm) and minimal inhibitory concentration (MIC in mg/ml) of hexanic, ethylacetate and methanolic extracts of five medicinal plants against Gram – bacteria species

Plant material	Extracts	***Proteus mirabilis***		***Escherichia coli***		***Shigella flexneri***		***Pseudomonas aeruginosa***		***Klebsiella pneumoniae***		***Salmonella typhi***	
	
		ID	MIC	ID	MIC	ID	MIC	ID	MIC	ID	MIC	ID	MIC
	
***Piptadeniastum africana***	Hexane	8	>20	0	>20	0	>20	10	20	10	>20	0	>20
	EtOAc	8	10	20	2.50	17	1.21	18	1.25	12	5	19	0.62
	MeOH	14	2.5	22	1.25	21	1.21	26	0.31	19	2.5	26	1.25
***Cissus aralioides***	Hexane	10	>20	11	>20	10	>20	12	20	10	>20	9	>20
	EtOAc	10	5	21	2.50	19	5	27	0.31	18	5	28	0.05
	MeOH	9	10	9	10	10	1.25	12	0.62	8	2.5	13	0.62
***Hileria latifolia***	Hexane	10	20	10	20	10	20	12	20	10	20	10	20
	EtOAc	15	1.25	15	1.25	15	1.25	25	0.62	15	2.50	24	0.62
	MeOH	13	1.25	19	1.25	18	1.25	26	0.62	16	2.50	24	0.62
***Phyllanthus muellerianus***	Hexane	10	10	10	20	13	20	14	10	10	10	13	10
	EtOAc	16	1.25	24	0.62	22	1.25	28	0.31	18	1.25	29	0.31
	MeOH	24	1.25	26	0.31	25	0.62	33	0.07	22	1.25	30	0.15
***Gladiolus gregasius***	Hexane	12	>20	11	>20	15	20	13	20	14	20	10	10
	EtOAc	10	10	12	10	14	20	13	10	12	10	13	5
	MeOH	0	>20	9	20	0	>20	0	>20	0	>20	12	10
Reference Drugs*		**14**	**0.12**	**16**	**0.12**	**17**	**0.03**	**15**	**0.06**	**15**	**0.06**	**22**	**0.06**

*Gentamycin was used as reference drug for bacteria species. ID: Inhibition zone diameters in mm; MIC: and minimal inhibition concentration. **Note: **Extracts and reference drugs were tested at the concentration of 50 mg/ml and 0.2 mg/ml respectively for the determination of IDs

**Table 3:** Inhibition zone diameters (ID in mm) and minimal inhibitory concentration (MIC in mg/ml) of hexanic, ethylacetate and methanolic extracts of five medicinal plants against Gram positive bacteria species and yeasts strains

PLANT MATERIAL	Extracts	Gram positive				Yeasts			
		
		***Staphylococcus aureus***		***Enterococcus faecalis***		***Candida albicans***		*Candida krusei*	
		
		ID	MIC	ID	MIC	ID	MIC	ID	MIC
		
*Piptadeniastum africana*	Hexane	0	>20	0	>20	9	20	12	20
	EtOAc	16	0.62	17	1.25	14	1.25	14	1.25
	MeOH	22	0.31	23	0.62	25	0.31	25	0.31
*Cissus aralioides*	Hexane	10	>20	10	>20	8	20	12	20
	EtOAc	23	0.31	21	1.2	21	1.25	20	1.25
	MeOH	9	10	10	5	12	0.62	10	0.62
*Hileria latifolia *	Hexane	12	20	12	20	11	10	10	5
	EtOAc	21	0.62	23	0.62	19	1.25	17	1.25
	MeOH	23	0.62	25	0.62	24	1.25	24	1.25
*Phyllanthus muellerianus*	Hexane	12	10	11	10	14	10	14	10
	EtOAc	23	0.31	25	0.31	23	0.62	23	0.62
	MeOH	26	0.31	28	0.07	28	0.31	28	0.07
***Gladiolus gregasius***	Hexane	13	20	9	20	12	20	12	10
	EtOAc	12	20	12	5	12	5	14	5
	MeOH	12	20	12	5	12	5	16	1.25
Reference Drugs*		25	0.06	17	0.06	22	0.01	20	0.02

*Gentamycin and Nystatin were used as reference drugs for bacteria species and yeasts strains respectively. ID: Inhibition zone diameters in mm; MIC: and minimal inhibition concentration. Note: Extracts and reference drugs were tested at the concentration of 50 mg/ml and 0.2 mg/ml respectively for the determination of IDs

*Cissus aralioides *had the highest inhibitory activity which was obtained with its EtOAc extract on *P. aeruginosa *(ID = 27 mm; MIC = 0.31 mg/ml) *and *on *S. aureus *(ID = 23 mm; MIC = 0.31 mg/ml). In general, all Gram negative bacteria species except *Proteus mirabilis *were sensitive to the 3 fractions of this plant, the most active fraction being the ethylacetate fraction. Amongst the Gram negative bacteria strains tested using the 3 fractions of *Hileria latifolia*, *P. aeruginosa *and *S. typhi *were considerably sensitive to both the ethylacetate (ID = 25 mm; MIC = 0.62 mg/ml) and the methanolic (ID = 26 mm; MIC = 0.62 mg/ml) fractions. The highest inhibitory activity on Gram positive strains was obtained with ethylacetate (23 ≤ID≤25 mm; 0.31≤MIC≤ 0.62 mg/ml) and methanolic (23≤ID≤25 mm; 0.31≤MIC≤ 0.62 mg/ml) fractions; its hexanic fraction induced only mild activity (10≤ID≤12 mm; 5≤MIC≤20 mg/ml). Ethylacetate and methanolic extracts of *Phyllanthus muellerianus *were found to be the most active fractions as compared to the hexanic. The methanolic and ethylacetate extracts of *Phillanthus muellerianus *showed the largest antimicrobial spectrum as they were active against all the microorganisms investigated with the most important ID (16 to 33 mm) and MIC (0.07 to 1.25 mg/ml). *P. aeruginosa, E. faecalis *and *C. krusei *(MIC = 0.07 mg/ml) were the most sensitive species to the MeOH extract whereas the hexanic fraction was less active.

Hexane, EtOAc and MeOH extracts of *G. gregasius *showed only mild activity compared to other plant'extracts and its methanolic extract was not active on *P*. *mirabilis, S. flexneri, P. aeruginosa *and *K. pneumonia*. The MIC values confirmed results obtained by the inhibition zone (Tables 2 and 3) ranging from 0.07 to >20 mg/ml.

In general, EtOAc and MeOH fractions of *Piptadeniastrum africana *and *Phyllantus muellerianus *happened to be the most active extracts with regard to their spectra of activity and the important inhibitory properties they induced.

### Acute toxicity

Acute toxicity was carried out on two medicinal plants: *Pitadeniastum africana *and *Phillanthus muellerianus. *They were selected based on their antimicrobial spectrum and efficacy. Because the ethylacetate and the methanolic extracts of these two plants were similar in terms of chemical composition and efficacy, the methanolic extracts of these two medicinal plants were used for toxicological evaluations; moreover these extracts were water soluble. During the observation period, no death of rats was neither recorded in the control nor in the treated groups. Visual observations of rats were made and recorded systematically 1 and 2 h after extract administration, including changes in respiratory, motility and skin sensitivity, diarrhea, behavioral pattern, weight gain, food and water consumption. These parameters changed mildly in groups 5 (16 g/kg bw) and 6 (20 g/kg bw) of male and female rats compared to the control. The pathological examination of the liver showed no visual abnormality in all groups at the end of the experiment.

Table 4 presents results of serum parameters for *Piptadeniastrum africana*. This extract administered at the dose of 4 g/kg bw showed no significant difference (P > 0.05) as compared to the treated group with regards to all the parameters tested. At the dose of 8 g/kg bw, an increased significant level (P < 0.05) of transaminases (ASAT and ALAT) was obtained, whereas serum creatinine and urea levels were not different from the control (P < 0.05). Administered at 12, 16 and 20 g/kg bw, all liver (ASAT and ALAT) and kidney (creatinine and urea) function tests increased significantly (P < 0.05 and P < 0.01).

**Table 4:** Effect of the methanolic extract of *Piptadeniastrum africana *on different sera biochemical parameters of male and female rats

***Dose(g/kg) ***	***Sex ***	***ALAT (UI) ***	***ASAT (UI) ***	***Creatinine (mg/l) ***	***Urea (mg/l)***
		
0	Male	74.60 ± 11.64	38.83 ± 0.18	9.00 ± 1.58	168.20 ± 1.58
	Female	74.50 ± 10.00	34.00 ± 4.21	10.22 ± 2.04	165.81 ± 0.42
4	Male	81.65 ± 2.92	38.66 ± 2.37	12.50 ± 4.21	162.20 ± 1.58
	Female	79.40 ± 2.15	37.28 ± 1.91	11.37 ± 4.32	158.11 ± 1.42
8	Male	89.65 ± 5.48*	44.20 ± 2.52*	11.25 ± 1.11	169.30 ± 1.22
	Female	88.10 ± 4.21*	42.32 ± 2.53*	12.70 ± 1.22	179.08 ± 1.91
12	Male	131.10 ± 1.73**	82.50 ± 1.52**	25.00 ± 2.53*	191.21 ± 2.00*
	Female	80.01 ± 2.10*	43.00 ± 1.21*	24.95 ± 2.14*	188.18 ± 1.45*
16	Male	154.25 ± 6.21**	93.41 ± 11.14**	36.36 ± 1.85**	214.21 ± 1.58**
	Female	124.01 ± 4.57**	92.58 ± 8.24**	40.15 ± 4.33**	195.82 ± 0.92**
20	Male	152.33 ± 6.23**	114.33 ± 6.12**	46.25 ± 2.25**	208.21 ± 1.18**
	Female	146.01 ± 3.27**	113.98 ± 3.21**	45.29 ± 5.41**	205.58 ± 1.25**

ANOVA *Significant difference between test groups and control at P < 0.05

** Significant difference between test groups and control at P < 0.01

Table 5 presents results of the effect of *Piptadeniastrum africana *on different liver biochemical parameters of male and female rats. As in Table 4, significant changes (P < 0.05 and P < 0.01) induced by this extract appeared only at high doses (8; 12; 16; 20 g/kg bw). An increased malondialdehyde was noted only at the dose of 8 g/kg bw, whereas, significant reduction in GSH level was obtained at 12 g/kg bw. Reduced levels of transaminases (ASAT and ALAT) in the hepatocytes were also observed at the dose of 8 g/kg bw.

**Table 5:** Effect of the methanolic extract of *Piptadeniastrum africana *on different liver biochemical parameters of male and female rats

***Dose (g/kg) ***	***Sex ***	***ALAT (UI/g of liver) ***	***ASAT (UI/g of liver) ***	***MDA (mmol/g of liver)***	***Glutathione (GSH)(μmol/g of liver) ***
		
0	Male	64.60 ± 10.34	40.83 ± 0.18	10.00 ± 1.58	4.12 ± 1.11
	Female	64.60 ± 09.00	40.00 ± 4.21	10.25 ± 2.04	4.62 ± 1.24
					
4	Male	61.66 ± 2.92	38.66 ± 2.37	9.50 ± 5.27	4.31 ± 2.23
	Female	65.50 ± 2.15	40.28 ± 1.95	9.97 ± 4.52	4.48 ± 1.21
					
8	Male	59.16 ± 3.48*	33.00 ± 1.82*	26.25 ± 3.44*	4.12 ± 1.11
	Female	58.20 ± 2.21*	30.12 ± 1.51*	25.70 ± 4.21*	4.62 ± 1.24
					
12	Male	21.00 ± 1.73**	30.50 ± 1.82**	45.00 ± 4.33**	2.32 ± 1.25**
	Female	53.00 ± 3.10*	37.00 ± 2.31*	24.95 ± 3.12*	3.16 ± 1.84*
					
16	Male	24.33 ± 3.21**	34.33 ± 12.14**	43.24 ± 2.85**	2.19 ± 1.23**
	Female	54.01 ± 3.57*	33.98 ± 8.24**	42.22 ± 5.41**	3.02 ± 1.34*
					
20	Male	19.33 ± 2.21**	24.33 ± 12.14**	41.15 ± 2.25**	2.31 ± 1.13**
	Female	21.01 ± 3.57**	23.98 ± 8.24**	40.25 ± 3.41**	2.22 ± 2.14**

ANOVA. *Significant difference between test groups and control at P < 0.05

** Significant difference between test groups and control at P < 0.01

Table 6 presents the effect of the methanolic extract of *Phyllanthus muellerianus *on different sera biochemical parameters of male and female rats. Sera biochemical parameters increased significantly only at doses starting from 8 g/kg bw. Sera transaminases increased markedly (P < 0.05) in male and female rats when the methanolic extract was administered at the dose of 8 g/kg bw. Administered at the dose of 4; 8; 12 g/kg bw, this extract had no significant effect on the levels of urea and creatinine but induced significant increase of these two parameters on the tested animals (P < 0.05 and P < 0.01) only at high doses (16 and 20 g/kg bw). Table 7 presents results of the effect of the methanolic extract of *Phyllanthus muellerianus *on different liver biochemical parameters of male and female rats. Significant decrease in hepatocyte transaminases (P < 0.05) as well as of GSH (P < 0.01) was only observed starting at 8 g/kg bw and 12 g/kg bw respectively. MDA serum level also significantly increased when administered at the dose of 8 g/kg bw.

**Table 6:** Effect of the methanolic extract of ***Phyllanthus muellerianus ***on different sera biochemical parameters of male and female rats

***Dose (g/kg) ***	***Sex ***	***ALAT (UI) ***	***ASAT (UI) ***	***Creatinine (mg/l) ***	***Urea (mg/l)***
		
0	Male	44.60 ± 11.64	41.83 ± 0.18	9.00 ± 1.58	157.21 ± 1.47
	Female	43.50 ± 10.00	42.00 ± 4.21	9.25 ± 2.04	155.81 ± 1.33
4	Male	41.66 ± 2.92	38.66 ± 2.37	7.50 ± 5.27	155.10 ± 1.18
	Female	42.50 ± 2.15	40.28 ± 1.95	7.27 ± 4.52	148.21 ± 1.22
8	Male	75.66 ± 5.48*	63.00 ± 1.82*	9.25 ± 2.44	159.40 ± 1.31
	Female	76.20 ± 4.21*	63.12 ± 1.51*	9.70 ± 2.21	169.18 ± 1.41
12	Male	86.00 ± 1.73*	160.50 ± 1.82**	9.10 ± 4.33	161.21 ± 1.60
	Female	76.90 ± 3.10*	160.01 ± 2.31**	9.55 ± 3.12	158.21 ± 1.25
16	Male	125.33 ± 6.21**	145.33 ± 12.14**	15.25 ± 2.85*	184.2 ± 1.58*
	Female	116.01 ± 3.57**	149.98 ± 8.24**	13.29 ± 5.41*	186.8 ± 0.92*
20	Male	124.33 ± 6.21**	144.33 ± 12.14**	25.25 ± 2.85**	219.24 ± 2.28**
	Female	123.01 ± 3.57**	123.78 ± 8.24**	21.29 ± 5.41**	215.58 ± 2.36**

ANOVA *Significant difference between test groups and control at P < 0.05

** Significant difference between test groups and control at P < 0.01

**Table 7:** Effect of the methanolic extract of ***Phyllanthus muellerianus ***on different liver biochemical parameters of male and female rats

***Dose (g/kg) ***	***Sex ***	***ALAT (UI/g of liver) ***	***ASAT (UI/g of liver) ***	***MDA (mmol/g of liver) ***	***Glutathione (GSH) (μmol/g of liver) ***
		
0	Male	74.60 ± 11.64	40.83 ± 0.18	10.00 ± 1.58	4.12 ± 1.11
	Female	74.50 ± 10.00	42.00 ± 4.21	10.25 ± 2.04	4.62 ± 1.24
					
4	Male	71.66 ± 2.92	42.66 ± 2.37	12.50 ± 5.27	4.31 ± 2.23
	Female	79.50 ± 2.15	40.28 ± 1.95	13.27 ± 4.52	4.48 ± 1.21
					
8	Male	79.36 ± 5.48*	43.00 ± 1.82*	16.25 ± 3.44*	4.12 ± 1.11
	Female	78.10 ± 4.21*	43.12 ± 1.51*	14.70 ± 4.21*	4.62 ± 1.24
					
12	Male	61.00 ± 1.73*	30.50 ± 1.82**	24.00 ± 4.33**	2.32 ± 1.25**
	Female	60.00 ± 3.10*	30.01 ± 2.31**	14.95 ± 3.12*	3.16 ± 1.84*
					
16	Male	44.33 ± 6.21**	24.33 ± 13.14**	26.15 ± 2.55**	2.19 ± 1.23**
	Female	44.01 ± 3.57**	23.98 ± 8.24**	23.21 ± 5.11**	3.02 ± 1.34*
					
20	Male	22.33 ± 6.21**	24.23 ± 10.14**	45.25 ± 2.15**	2.31 ± 1.13**
	Female	24.01 ± 3.57**	22.68 ± 6.24**	46.29 ± 5.21**	2.22 ± 2.14**

ANOVA *Significant difference between test groups and control at P < 0.05

** Significant difference between test groups and control at P < 0.01

## Discussion

Successful prediction of chemical compounds from plant material is largely dependent on the type of solvent used in the extraction procedure. The traditional healers or practitioners make use of water primarily as a solvent. In this study, extraction was done using organic solvent such as hexane, methanol and EtOAc. This is because these solvents are easily evaporated and permit an easier estimation of extract concentration, which is difficult to obtain with water as solvent. Results of the analysis of five medicinal plants extracts using these solvents showed that their chemical content differs depending on the nature of solvent used. Identical classes of chemical compounds are found in the hexanic, methanolic and EtOAc extracts of *P. africana*; however, flavonoids, polyphenols, cardiac glycosides and reducing sugars were mostly found in the methanolic and EtOAc fractions. Alkaloids were absent in the methanolic fraction and this could be justified by its poor polarity. Both Gram negative and Gram positive were sensitive to ethylacetate and methanolic fractions of the five medicinal plants rich in flavonoids, polyphenols and anthraquinons. This is probably due to their ability to complex with extracellular and soluble proteins and to complex with bacterial cell walls; more lipophilic flavonoids may also disrupt microbial membranes, [23,24]. The presence of coumarins in the various fractions of *P. muellerianus*, *H. latifolia*, *C. aralioides *and *P. africana*, may explain their important inhibitory activity against *Candida albicans *and *Candida krusei. *This anticandidal activity of coumarins has been demonstrated *in vitro *and *in vivo *against vaginal candidiadis by Thornes [25]. Success of extracts from *P. africana, C. aralioides and G. gregasius *against both Gram positive and Gram negative agents is likely dependent on their content in alkaloids able to intercalate between DNA strands [26]. The presence of sterols and triterpens has been confirmed in another study carried out in Cameroon by Mbouangouere *et al. *[27] who isolated terpenes such as sitosterol, β-amyrin and eicosane; however they found no alkaloids in this plant. The absence of alkaloids in their study could be due to the fact that, their investigation was done on a defatted fraction of the methanol/CH_2_Cl_2 _extract. The hexanic fraction induced only mild activity compared to the ethylacetate and methanolic fractions to which all microbial strains tested were sensitive. This could indicate that important bioactive principles of *Piptadeniastrum africana *are of polar nature. Polar secondary metabolites like flavonoids, coumarins, anthraquinons, phenols and glycosides have been found to possess important antimicrobial properties [5,28]. In accordance, Mbouangouere *et al. *[27] obtained inhibitory activity on Gram positive and Gram negative strains (*Escherina coli, Bacillus subtilis, Shigella flexenari, Staphylococcus aureus, Pseudomonas aeruginosa, Salmonella typhi) *with ID varying between 9-16 mm when tested at 3 mg/ml. In the present study, we obtained a similar activity with *P. africana *against *S. aureus, P. aeruginosa, C. albicans and C. krusei *with ID varying between 14 and 26 mm. In a previous epidemiological study, these microorganisms were found to be highly resistance to ampicillin and augmentin [6]. With regards to these results we can deduce that this plant' extract, *P. africana*, is a good source of antimicrobial agents with interesting activity on multi resistant strains.

Looking at it toxicity parameters, no death of rats was neither recorded in the control nor in treatment groups during acute toxicity indicating an LD_50 _> 5 g/Kg bw. From these results, it can be concluded that *P. africana *is not toxic with regard to the threshold of toxic substances (5 g/kg) as previously stipulated [15-17].

Results of sera and liver biochemical parameters indicated that the variation of transaminase (ALT and AST) activities are associated with hepato-cellular damage, although marked increased in these parameters were only observed at a higher dosage (≥8 g/kg bw). The concurrent significant increase of ALT and AST activities in sera and decrease ALT and AST activities in hepatocytes after treatment of the male and female rats above 8 g/kg bw clearly indicate leakages of liver cell contents into the blood stream. The creatinine and urea levels of treated rats increased significantly as compared to the control only at doses above 12 g/kg bw. This result confirms that the rats' kidneys were affected only when administered high quantity of the extract (≥8 g/kg bw). The variation of other parameters such as glutathione and malondialdehyde was also significant only at large doses (≥8 g/kg bw) indicating that lipid peroxidation started earlier and resulted in oxidative stress as reflected by the increased GSH depletion and MDA levels in rats hepatocytes.

The presence of alkaloids, triterpens, sterols, tannins, coumarins, glycosides, cardiac glycosides and reducing sugars in the three fractions of *Cissus aralioides *could account for the important antimicrobial activity exhibited by this plant against all the tested microorganisms.

The antimicrobial property of *Hileria latifolia *has not been revealed before; only few studies have indicated the presence of alkaloids and flavonoids in Ivory Coast [29]. The species investigated in this study shows that it is instead rich in triterpens, sterols and coumarins, in addition to saponins, glycosides and reducing sugars found in the methanolic fraction of the plant. The important antimicrobial property of the plant in the ethylacetate fraction may be due to the presence of these metabolites [30-34]. Previous studies had shown that this plant when administered orally at a low dosage in rats is less toxic [35].

Mild antibacterial and antifungal activities (on *Candida albicans *and *Candida krusei) *of *Gladiolus gregasius *observed in this study are in contrast with previous work done by Assob *et al. *[36] who found that the bulb extract was fungicidal with no antibacterial activity. The difference may be due to harvesting periods and solvent used in the extraction of the plant [37]. The hydroethanolic extract of the plant was found not to induce toxicity in rats [36].

An important antimicrobial activity was observed with the three fractions of *Phyllantus muellerianus *against Gram positive and Gram negative bacteria species and as well as against *Candida *species. These results are in concordance with the work carried out by Doughari and Sunday [38] who obtained similar activity against some pathogenic bacteria. However, the species tested in this study seem to be more active considering its inhibitory properties (Highest ID = 30 mm, MIC = 0.15, vs. ID = 18 mm, MIC = 625 μg/ml in their study) and to possess a larger spectrum of activity against tested microorganisms. The phytochemical screening showed almost the same chemical composition (tannins, flavonoids, polyphenols, saponins, alkaloids, and anthraquinones), except that alkaloids were absent in our study. Another study revealed the antitimicrobial properties of the methanolic extract of the stem bark of *Phyllanthus muellerianus *against *Clostridium sporogenes*, *Streptococcus mutans *and *Streptococcus pyogenes *[39]. *P. muellerianus *presented no adverse effect on the liver and kidney functions when administered at a low dosage (4 g/kg bw) to male and female rats in this study. Based on this, it can be concluded that *P. muellerianus *is not toxic, taking into consideration the threshold of toxic substances (5 g/kg) as earlier stipulated [16-18]. No previous toxicological evaluation has been carried out on this plant. But, works done on *Phyllantus sp. *have revealed their hepatoprotective, antiulcerative, gastroprotective and antiviral properties which may probably justify the innocuity of the plant [40-43]. The occurrence of decreased levels of GSH and increased MDA respectively when administered at elevated dosage (8 and 12 g/kg bw respectively) shows that this extract contains bioactive hepatoprotective agents whose efficacy is only exceeded when high concentration of the extract is absorbed. The liver injuries due to this extract is minimized and may be due to the enhancement of hepatic glutathione regeneration capacity and the decreased level of lipid peroxidation, particularly when moderate doses of the extract are absorbed (4 g/kg bw). This protective effect is also associated with the presence of flavonoids and triterpens in the stem bark of the plant conferring it, an important antioxidant property [44]. Experiments conducted at the Niwa Institute of Immunology in Japan have shown flavonoids in *E. officinalis *to be potent scavenger of free radicals [45] and triterpenoids to play an essential role in the protection of the liver against free radicals. Triterpenoids were shown to be able to protect mice against carbon tetrachloride (CCl4)-induced hepatotoxicity, and inhibit lipid peroxidation in rat liver microsomes [46-48].

## Conclusions

In general, the highest antimicrobial activities were obtained with *Phyllanthus muellerianus *and *Pitadeniastum africana *extracts. However, all the five medicinal plants, *Pitadeniastum africana, Cissus aralioides, Hileria latifolia, Phyllantus muellerianus *and *Gladiolus gregasius *tested are antibacterial and anticandidal agents and could be used in the treatment of various urogenital and gastrointestinal ailments caused by multiresistant microbial agents. It is also clear from this study that, the antimicrobial activity of these five medicinal was mainly found in the methanolic and ethylacetate fractions. The antimicrobial activities of *Cissus aralioides *and *Hilaria latifolia *have not been studied before. This study has also revealed that *Piptadeniastrum africana *and *Phyllantus muellierianus *are not toxic at therapeutic doses with good antimicrobial properties. This study is an important step towards clinical evaluation in order to produce improved phytomedicine in the treatment of multiresitant microbial infections. Alongside further chemical analysis involving CG-MS, HPLC-MS or ESI-MS analysis will enhance our comprehension on the nature of the chemical compounds contain in the extracts.

## Competing interests

The authors declare that they have no competing interests.

## Authors' contributions

JCNA as principal investigator designed and implemented the project and initiated the writing of the manuscript. VBP assisted in designing the project and revised the manuscript. HLFK assisted in the *in vitro *antimicrobial testing and in revising the paper. DSN assisted in the identification of microbial strains, the statistical analysis of data and in revising the paper. ALN provided technical assistance during antimicrobial testing. PFN assisted in the identification of the microbial strains used and contributed in the write up. EAA assisted in designing the project. AJN carried out the antimicrobial and toxicological tests. BS contributed in the collection, identification and extraction of plant material and authorized the *in vivo *acute toxicological investigations in the Phytorica Lab in Douala. All the authors read and approved the final manuscript.

## Pre-publication history

The pre-publication history for this paper can be accessed here:

http://www.biomedcentral.com/1472-6882/11/70/prepub
